# GLCCI1 reduces collagen deposition and airway hyper‐responsiveness in a mouse asthma model through binding with WD repeat domain 45B

**DOI:** 10.1111/jcmm.16658

**Published:** 2021-05-28

**Authors:** Qiufen Xun, Jiulong Kuang, Qing Yang, Wei Wang, Guofeng Zhu

**Affiliations:** ^1^ Department of Respiratory and Critical Care Medicine Second Affiliated Hospital of Nanchang University Nanchang China

**Keywords:** asthma, autophagy, glucocorticoid‐induced transcript 1 gene, WD repeat domain 45B

## Abstract

Asthma is a serious public health problem worldwide, without effective therapeutic methods. Our previous study indicated that glucocorticoid‐induced transcript 1 gene (GLCCI1) knockout reduces the sensitivity to glucocorticoid in asthmatic mouse. Here, we explored the role and action mechanism of GLCCI1 in asthma development. In ovalbumin‐sensitized mice, airway resistance and tissue damage increased, the production of inflammatory cytokines were up‐regulated, GLCCI1 expression was reduced and autophagy was activated. Increasing of GLCCI1 inhibited human and mouse airway epithelial cell (AEC) autophagy, while decreasing of GLCCI1 promoted autophagy. Furthermore, we found that GLCCI1 bound with WD repeat domain 45B (WDR45B) and inhibited its expression. Increasing of WDR45B partly reversed the inhibition of GLCCI1 to autophagy‐related proteins expression and autophagosome formation in vitro. Increasing of WDR45B in vivo reversed the improvement of GLCCI1 on airway remodelling in asthma and the inhibition to autophagy level in lung tissues. Overall, our data showed that GLCCI1 improved airway remodelling in ovalbumin‐sensitized mice through inhibiting autophagy via combination with WDR45B and inhibiting its expression. Our results proved a new idea for asthma treatment.

## 1 INTRODUCTION

Asthma is a serious public health problem globally, and the major types of it include allergic asthma, non‐allergic asthma, late‐onset asthma, fixed airflow‐related asthma and obesity‐related asthma. In accordance with the data from the World Health Organization, about 235 million people are affected by asthma worldwide.^1^ Asthma is a heterogeneous disorder caused by various factors, such as allergies and air pollution, and is closely associated with inflammation and airway constriction.^2^, ^3^ Currently, although many drugs were applied for asthma treatment, the outcome of asthma treatment still is unsatisfactory.^4^ Glucocorticoid‐induced transcript 1 gene (GLCCI1) was first described as a thymocyte‐specific transcript; the polymorphism of it was proved to be involved in the response to inhaled corticosteroids in asthmatic patients.^5^, ^6^ Some previous evidence has indicated that the polymorphisms of *GLCCI1* are associated with the airway hyper‐responsiveness.^7^ In our previous study, we found that expression of GLCCI1 is associated with the changes of forced expiratory volume in 1 second in asthmatic patients accepted with fluticasone propionate treatment.^8^ Moreover, in our previous study, we also demonstrated that knockout *GLCCI1* in asthmatic mice could attenuate the therapeutic outcome of hydroprednisone.^8^ Above studies have suggested that GLCCI1 may play a crucial role in the development of asthma, while the mechanism of how GLCCI1 influences asthma development remains unclear.

Autophagy is a classical self‐degradative pathway existed in eukaryotic cells, which can be divided into macroautophagy, microautophagy and chaperone‐mediated autophagy.^9^ It was well known that autophagy is a complex process, mediated by a series of proteins, including microtubule‐associated protein 1 light chain 3 (LC3), autophagy‐related genes (ATGs), Beclin‐1 and p62, and normal autophagy plays a crucial role in cell homeostasis.^10^, ^11^ A growing evidence has reported that autophagy involved in the development of asthma, it is a potential target for asthma therapy.^12^ Activated autophagy was found in the large and small airway epithelium of the patients with asthma, as well as in transforming growth factor β (TGF‐β)–stimulated airway smooth muscle cells, and involved in the airway remodelling in mouse asthma model.^13^, ^14^ Given the hypothesized link between asthma pathogenesis and autophagy, we sought to identify whether the autophagy‐promoting gene products of WD repeat domain 45B (WDR45B) were involved in GLCCI1 modulation of asthma susceptibility and treatment response.

WDR45B is a homolog of ATG8, also named as WIPI3; the deficiency of it was proved to be a reason for neuronal apoptosis defection.^15^ WDR45B binds with tuberous sclerosis complex to promote autophagy.^16^ In this present study, we found that GLCCI1 inhibited the airway remodelling in ovalbumin‐sensitized mice through suppression of the autophagy of airway epithelia cells (AECs) via binding with WDR45B. Our data reported a novel pathogenesis of asthma and provided a new potential target for asthma treatment.

## 2 MATERIALS AND METHODS

### 2.1 Animals and mouse asthma model

Wild‐type C57BL/6 mouse (female, 6‐ to 8‐week‐old, 18‐20 g) was purchased from Liaoning Changsheng Biotechnology Co., Ltd. All mice were raised in a specific pathogen‐free environment and given enough food and water. All experiments were carried out strictly in accordance with the Guideline for the Care and Use of Laboratory Animals. Meantime, all animal experiments were approved by the institutional committee of the institute.

Mouse asthmatic model was established as our previous study.^8^ On day 1, day 7 and day 14, the mouse was intraperitoneally injected with 50 μg of chicken ovalbumin (Aladdin) and 2 mg of aluminium hydroxide (Sigma). Ovalbumin and aluminium hydroxide were co‐dissolved in 0.2 mL sterile saline. The mouse in control group was intraperitoneally injected with only sterile saline. From day 21 to day 27, mouse asthma model was exposed to 5% ovalbumin for 30 minutes, once a day. Control mice were exposed to sterile saline. Lentivirus (HANBIO) containing GLCCI1‐overexpressed plasmid, WDR45B‐overexpressed plasmid or negative control (3 × 10^6^ infectious units) were intratracheally injected into the ovalbumin‐sensitized mice at three days before the first ovalbumin challenge. At 24 hours after the last airway challenge, airway resistance of mouse was measured as previous study using a computer‐controlled flexiVent system (SciReq).^17^ Bronchoalveolar lavage fluid (BALF) and serum in mice were collected, and other experiments were carried out.

### 2.2 Cell culture and treatment

BEAS‐2B, a human bronchial epithelial cell line, was obtained from American Type Culture Collection (ATCC). All cells were cultured in a standard condition, 5% CO_2_ and 37°C temperature. Dulbecco's modified Eagle's medium/F12 medium (Invitrogen), supplemented with 10% foetal bovine serum (FBS, Invitrogen), 100 ng/mL streptomycin and 100 U/mL penicillin, was utilized to culture BEAS‐2B cells. In cellular experiments, 300 ng/mL of tumour necrosis factor α (TNF‐α) was used to stimulate AECs. AECs were infected with the lentivirus containing GLCCI1‐overexpressed plasmid or its negative control, GLCCI1 shRNA (shGLCCI1) or negative control (shRNA), WDR45B‐overexpressed plasmid or its negative control before TNF‐α stimulation.

### 2.3 Measurement of IL‐1β, TGF‐β and TNF‐α concentration

At 24 hours after the last airway challenge, serum and BALF samples were collected from control mice and ovalbumin‐sensitized mice. Blood was collected from the retro‐orbital space under anaesthesia. Then, the blood was centrifuged at 300 *g* for 15 minutes at 4°C for serum obtaining. For collection of BALF samples, the mice were anaesthetized using 10% chloral hydrate (0.5 mg/kg) by intraperitoneal injection. Then, tracheas were inserted with a catheter by the way of an incisal opening sited in the cervical part, and airway lumina were washed using PBS. Subsequently, concentration of pro‐inflammatory cytokines, IL‐1β, TNF‐α and TGF‐β, in BALF and serum were measured by ELISA. Mouse IL‐1β ELISA kits, mouse TGF‐β ELISA kit and mouse TNF‐α ELISA kit were purchased from R&D Systems. All experiments were carried out strictly according to the manufacture's introduction.

### 2.4 Detection of inflammation and collagen deposition in lung tissues of mice

H&E staining was performed to assess pathological changes in lung tissues. At 24 hour after the last ovalbumin challenge, all mice were killed, and lung tissues were removed. Lung tissues were dehydrated, and then were embedded into paraffin. Next, 4‐μm thick sections were used for H&E staining. Sections were successively stained with eosin and haematoxylin (Solarbio) according to the kit protocol. Then, a light microscope was used to take pictures. Inflammatory cell infiltration degree: 0: no inflammatory cell infiltration; 1: a small number of infiltrated inflammatory cells; 2: an inflammatory cell monolayer around the airway; 3: inflammatory cell bilayer around the airway; 4: four layers of inflammatory cells around the airway. In addition, Masson staining was performed to detect the deposition of collagen in lung tissues of mice. Masson staining kit was obtained from Solarbio, and experiment was fulfilled according to the appropriate introduction. Image‐Pro Plus 6 software was used to analyse the area of collagen‐positive. Collagen volume fraction (CAV/%) = (area of collagen − positive/total area of the tissues) × 100%.

### 2.5 Examination of proteins expression

Expression of GLCCI1, Beclin‐1, LC3‐II, LC3‐I, p62, WDR45B, Collagen I (COL I), COL III, MMP‐9 and TIMP‐1 was measured by Western blot. Firstly, total protein was isolated from lung tissues and cells using RIPA lysis buffer (Servicebio). Then, an equal quantity (20 μg) of each samples was separated on 12% SDS‐PAGE and then was transferred onto polyvinylidene fluoride membranes (Millipore, IPVH00010). After that, all membranes were maintained with 5% non‐fat milk for 1 hour at room temperature. All membranes were incubated with primary antibodies working solution (Abcam, 1:2000 dilution) at 4°C overnight. Next day, all membranes were incubated with secondary antibodies for 1 hour at room temperature. Finally, an ECL kit (Sigma) was used to display protein bands. Relative expression of proteins was analysed using ImageJ software. LC3, Beclin‐1 and p62 are protein markers of autophagosome formation.

### 2.6 Detection of autophagy in lung tissues

Immunocytochemistry assay was performed to detect the expression of LC3 in lung tissues for the analysis of autophagy. Paraffin‐embedded lung sections were incubated with 3% H_2_O_2_ to inhibit endogenous peroxidase activities, 15 minutes at room temperature. Next, sections were maintained with normal goat serum for 15 minutes at room temperature followed by primary antibody against LC3 at 4°C overnight. After that, sections were incubated with secondary antibody (1:500, Beyotime) for 1 hour at 37°C. Subsequently, the sections were stained with DAB (Solarbio) in accordance with the kit introduction, and then, nucleuses were marked with DAPI solution. A confocal microscope was used to observe the results.

### 2.7 Examination of mRNAs expression

GLCCI1 mRNA level in lung tissues was measured using qRT‐PCR assay. Total RNA was isolated from tissues utilizing TRIzol reagent (Invitrogen), followed by cDNA synthesis using a RevertAid First Strand cDNA Synthesis Kit (Thermo Fisher Scientific). Next, real‐time PCR was performed on an ABI VIIA 7 real‑time PCR system (ABI) according to the introduction of SYBR Premix Ex Taq II (TaKaRa). Relative expression of GLCCI1 mRNA was analysed in accordance with 2^−ΔΔCt^ method and normalized to *GAPDH*.

### 2.8 Detection of autophagy in AECs

The number of autophagosome in AECs was examined using immunofluorescence analysis. After infection, the AECs were washed with pre‐cold PBS buffer, and then were fixed with 4% paraformaldehyde for 30 minutes at 4°C. Then, AECs were permeabilized with 0.2% Triton X‐100 in PBS at room temperature for 15 minutes. Subsequently, AECs were incubated with the antibody of LC3 at 4°C overnight. Next, AECs were maintained with Alexa Fluor 488 goat anti‐rabbit secondary antibody (Invitrogen). The formation of autophagosome was observed under a confocal laser scanning microscope (Leica), and the number of it was counted.

### 2.9 Analysis of GLCCI1 binds with WDR45B

Co‐immunoprecipitation was carried out to detect the relationship between GLCCI1 and WDR45B in AECs. Human and mouse AECs were lysed according to the previous study.^18^ Then, 2 mg of cell lysates were incubated with protein G beads (Pierce Crosslink IP Kit, Thermo Fisher Scientific) at 4°C for 30 minutes, and protein G beads pre‐bound with antibodies of GLCCI1 and WDR45B (Abcam) at 4°C overnight. Subsequently, the protein complex was washed from beads using glycine, and the expression of WDR45B and GLCCI1 was measured by Western blot.

### 2.10 Statistical analysis

In our study, the software of SPSS 20.0 and GraphPad Prism 6.0 was used to analyse the data and make images, respectively. All data were displayed as mean ± standard deviation (SD). The significant difference between two independent groups was analysed using Student's *t* test. One‐way ANOVA followed by Tukey post hoc test was used to the analysis of multiple groups. *P* < .05 was considered statistical difference.

## 3 RESULTS

### 3.1 GLCCI1 was decreased and autophagy was activated in ovalbumin‐sensitized mice

**FIGURE 1:**
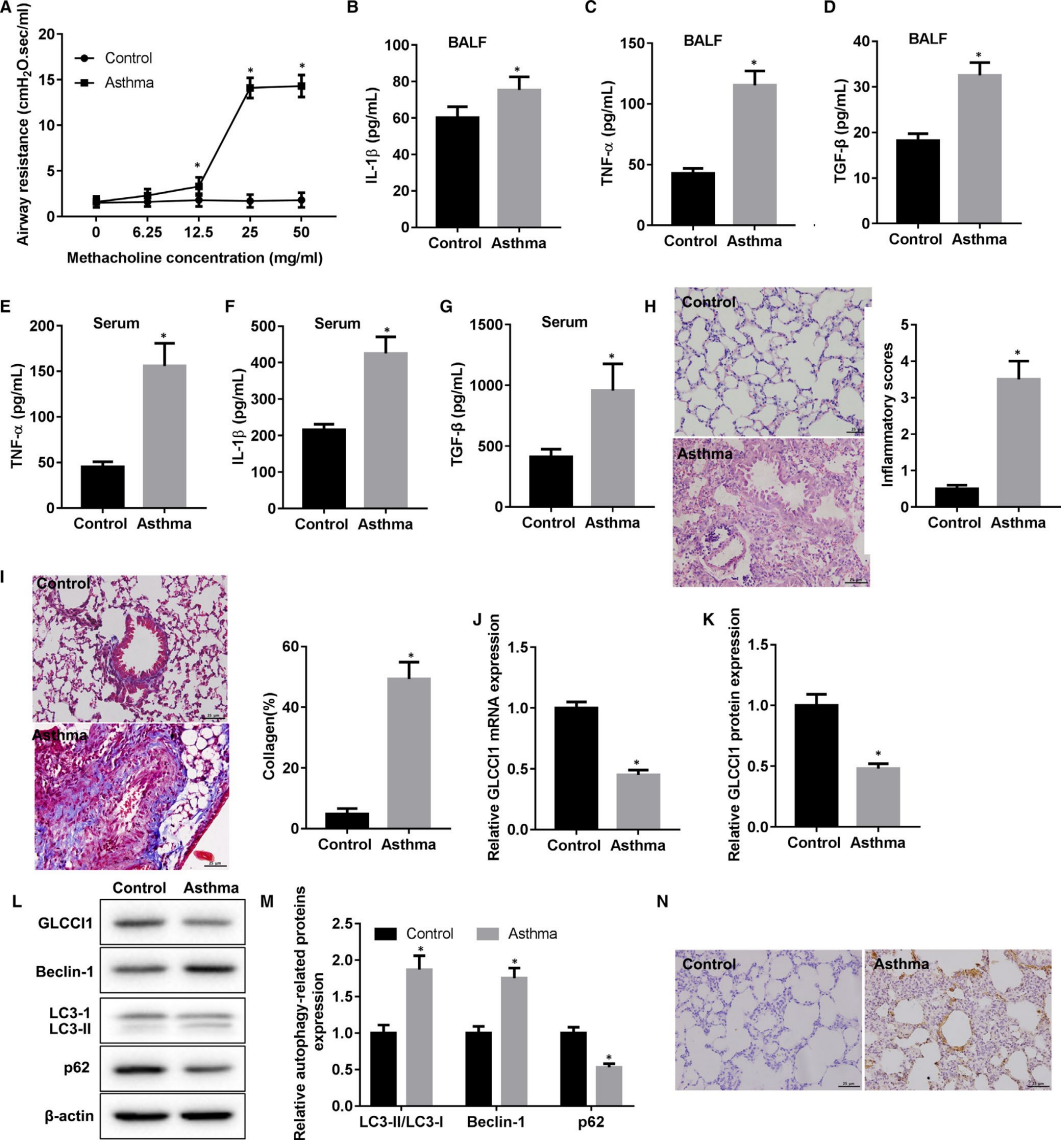
GLCCI1 in ovalbumin‐sensitized mice was decreased and autophagy was activated. A, Ovalbumin‐sensitized mice and control mice were stimulated with methacholine at different doses, and then, the airway resistance of them was examined. B‐D, The concentration of IL‐1β, TNF‐α and TGF‐β in BALF of all mice was detected using ELISA. E‐G, The concentration of TNF‐α, IL‐1β and TGF‐β in serum of all mice was also measured by ELISA. H, H&E staining was performed to detect the pathological changes in lung tissues of ovalbumin‐sensitized mice. I, Masson staining was carried out to examine the deposition of collagen fibre in lung tissues of mice. J, The expression level of GLCCI1 mRNA in lung tissues of mice was examined by qRT‐PCR. K‐M, The expression levels of GLCCI1, Beclin‐1, LC3‐II/I and p62 protein in lung tissues of mice were measured by Western blotting assay and analysed. N, Immunocytochemistry was used to analyse the level of autophagy in lung tissues through detection of LC3‐positive cells. N = 6 mice per each group. *P < .05 compared with control group

In this present study, our data presented that airway resistance in ovalbumin‐sensitized mice significantly higher than control mice following they were treated with methacholine at an increasing doses from 12.5 to 50 mg/mL (Figure 1A). Several highly expressed inflammatory cytokines, including IL‐1β (Figure 1B), TNF‐α (Figure 1C) and TGF‐β (Figure 1D), were examined in BALF of ovalbumin‐sensitized mice. Meantime, the concentration of TNF‐α (Figure 1E), IL‐1β (Figure 1F) and TGF‐β (Figure 1G) was also found to increase in the serum of ovalbumin‐sensitized mice. As shown in Figure 1H, the lung tissues from ovalbumin‐sensitized mice displayed obviously airway remodelling, manifested by, such as thickening of airway smooth muscle. Besides, we further proved that excessive collagen accumulated under the airway epithelium in ovalbumin‐sensitized mice (Figure 1I). Importantly, our results demonstrated that the expression of *GLCCI1* was notably suppressed in the lung tissues of ovalbumin‐sensitized mice (Figure 1J). GLCCI1 protein expression also was obviously inhibited in ovalbumin‐sensitized mice (Figure 1K,L). The expression of autophagy‐related protein, p62, was down‐regulated in the lung tissues of ovalbumin‐sensitized mice. However, the expression of autophagy‐related proteins, including LC3 II/I and Beclin‐1, was facilitated in lung tissues of ovalbumin‐sensitized mice (Figure 1L,M). Immunohistochemical detection of LC3 shown that the level of autophagy was markedly increased in lung tissues from ovalbumin‐sensitized mice compared with that from control mice (Figure 1N). Overall, we successfully obtained the mice asthma model. We found that GLCCI1 expression in ovalbumin‐sensitized mice was restrained, and autophagy was activated.

### 3.2 GLCCI1 expression was positively associated with low autophagy level in asthma

**FIGURE 2:**
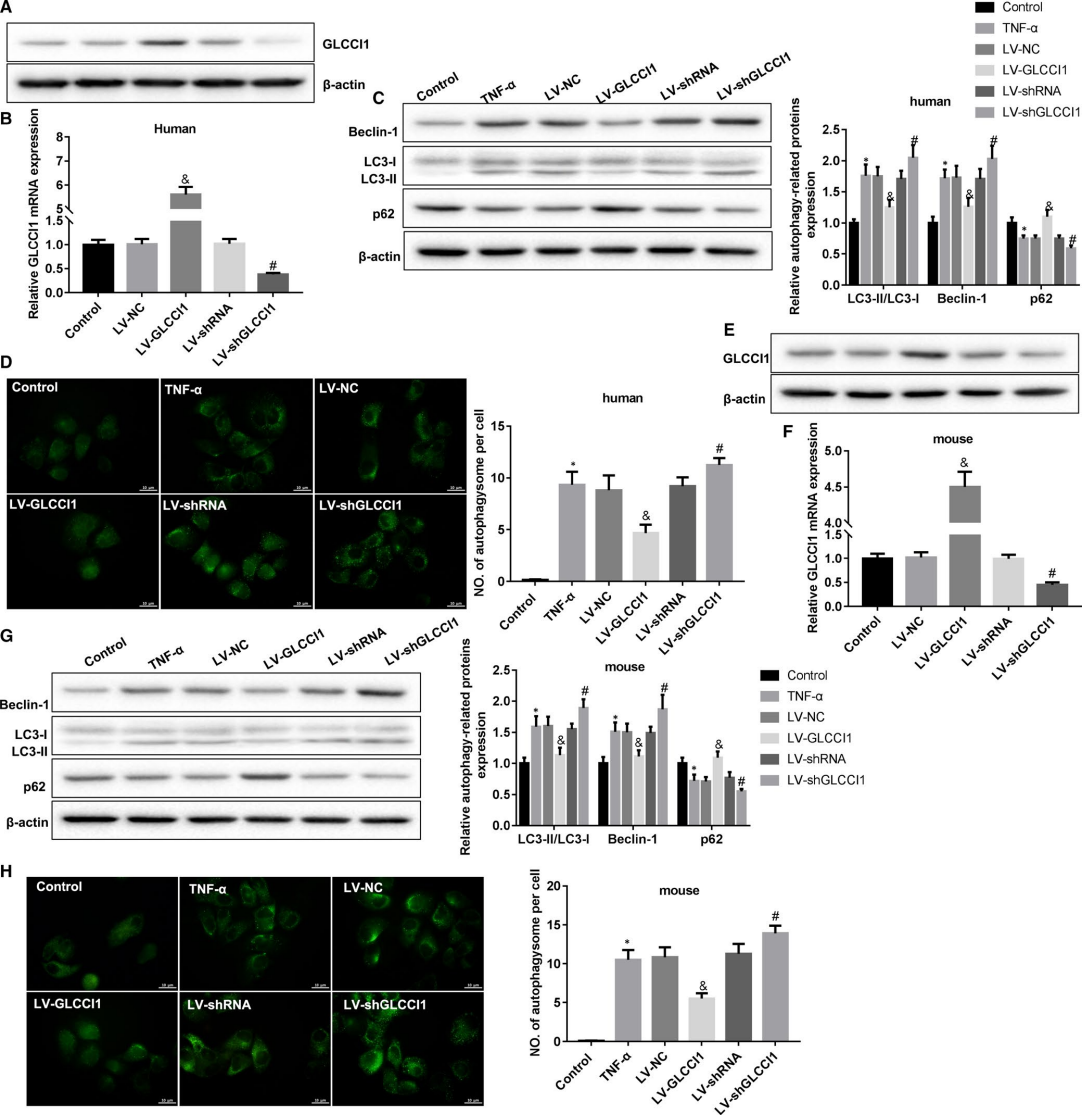
Effect of GLCCI1 on activated autophagy in human and mouse AECs. A and E, Expression of GLCCI1 protein in the human and mouse AECs infected with lentivirus was measured by Western blotting assay to verify the interference efficiency. B and F, Expression of GLCCI1 gene in human and mouse AECs infected with lentivirus was examined by qRT‐PCR to verify the interference efficiency. C and G, Western blotting assay was carried out to detect the expression of autophagy‐related factors in human and mouse ACEs, including Beclin‐1, LC3‐II, LC3‐1 and p62. D and H, The formation of autophagosome in human and mouse AECs was examined utilizing immunofluorescence assay. N = 3. *P < .05 compared with control group, ^&^ *P* < .05 contrasted with LV‐NC group and ^#^ *P* < .05 compared with LV‐shRNA group

To explore the effect of GLCCI1 on autophagy in ovalbumin‐sensitized mice, we cultured human and mouse AECs in vitro, which infected with lentivirus carrying empty vector, GLCCI1 overexpression plasmid, shRNA negative control or shGLCCI1. As shown in Figure 2A,B, in human AECs, the expression of GLCCI1 mRNA and protein was obviously promoted by LV‐GLCCI1 and inhibited by LV‐shGLCCI1. Furthermore, in TNF‐α‐stimulated human AECs, LC3‐II/I and Beclin‐1 expression were up‐regulated, while p62 expression was down‐regulated. Overexpression of GLCCI1 notably reduced the expression of LC3‐II/I and Beclin‐1 and enhanced p62 expression. Knockdown of GLCCI1 expression could boost LC3‐II/I and Beclin‐1 expression and suppress p62 expression (Figure 2C). TNF‐α promoted the formation of autophagosome in human AECs. Increasing of GLCCI1 could markedly reduce the numbers of TNF‐α‐induced autophagosome, and knockdown of GLCCI1 increased TNF‐α‐induced autophagosome numbers (Figure 2D). In mouse AECs, we also demonstrated that GLCCI1 mRNA and protein expression were facilitated by LV‐GLCCI1 and suppressed by LV‐shGLCCI1 (Figure 2E,F). In TNF‐α‐stimulated mouse AECs, the expression of LC3‐II/LC3‐I and Beclin‐1 was also facilitated, and p62 expression was inhibited. Overexpression of GLCCI1 could effectively decrease the expression of LC3‐II/LC3‐I and Beclin‐1 and increase p62 expression, and down‐regulation of GLCCI1 resulted an opposite effect on these factor expression (Figure 2G). Moreover, increasing of GLCCI1 could also repress TNF‐α‐induced autophagosome in mouse AECs, and decreasing of GLCCI1 promotes TNF‐α‐induced autophagosome (Figure 2H). In summary, overexpression of GLCCI1 effectively inhibited TNF‐α‐induced autophagy in AECs, and knockdown of GLCCI1 significantly facilitated TNF‐α‐induced autophagy.

### 3.3 GLCCI1 suppressed human AECs autophagy through binding to WDR45B

**FIGURE 3:**
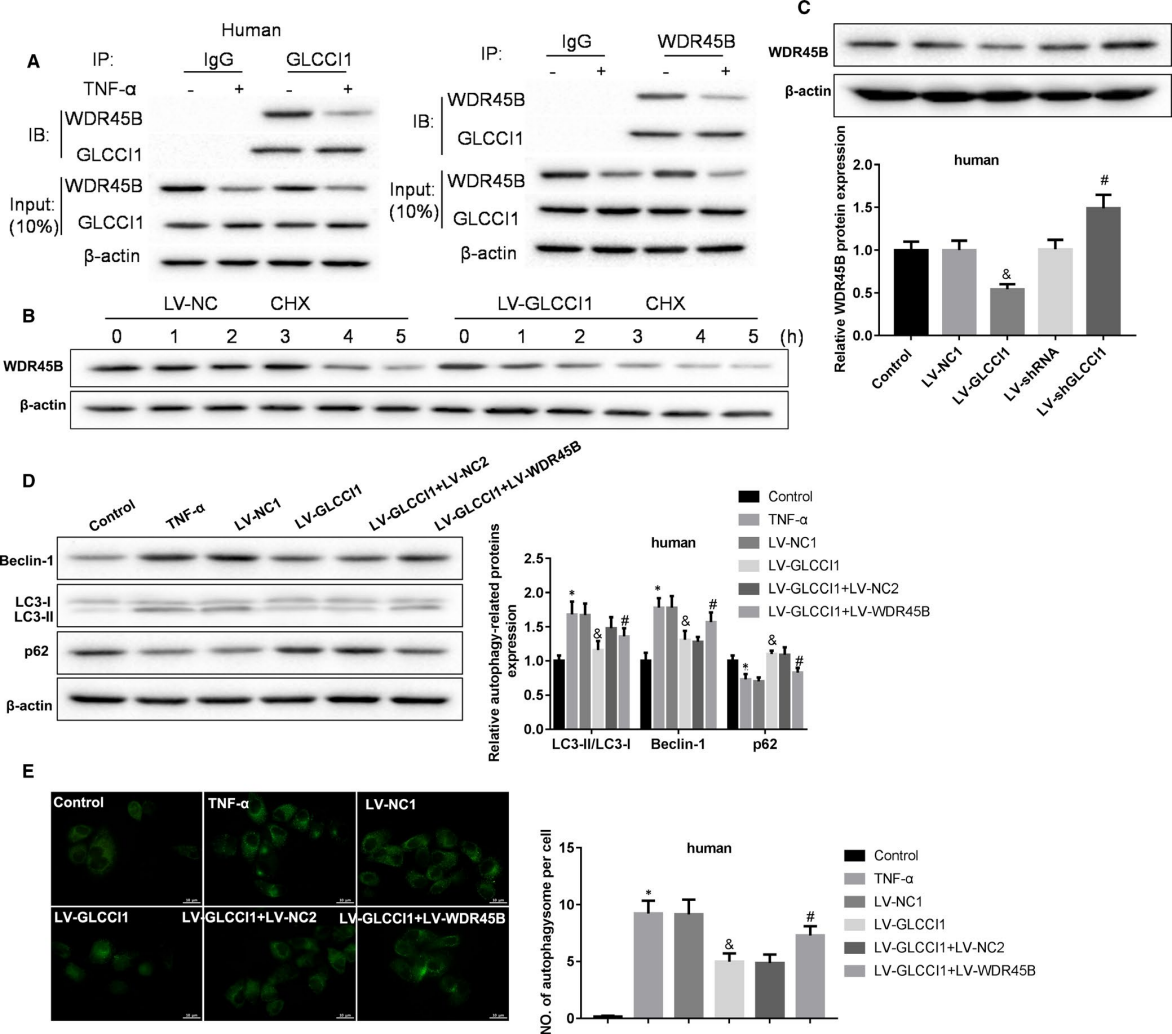
GLCCI1 inhibited TNF‐α‐induced human AECs autophagy by binding with WDR45B and suppression its expression. A, Co‐IP experiments were carried out to examine the combination between GLCCI1 and WDR45B. B, 50 mg/mL of CHX was used to stimulate human AECs infected with LV‐NC or LV‐GLCCI1 for 0, 1, 2, 3, 4 and 5 h. Then, the expression of WDR45B in cells was measured by Western blotting assay. C, Western blotting assay was performed to examine the expression of WDR45B in human AECs infected with LV‐GLCCI1 or LV‐shGLCCI1. N = 3. ^&^ *P* < .05 contrasted with LV‐NC1 group, and ^#^ *P* < .05 compared with LV‐shRNA group. D, Western blotting assay was implemented to detect the expression of Beclin‐1, LC3‐II, LC3‐I and p62 in human AECs co‐infected LV‐GLCCI1 with or without LV‐WDR45B. E, Formation of autophagosome was measured using immunofluorescence assay. N = 3. *P < .05 compared with control group, ^&^ *P* < .05 contrasted with LV‐NC1 group and ^#^ *P* < .05 compared with LV‐GLCCI1 group

Next, we investigated the regulatory mechanism of GLCCI1 in TNF‐α‐induced human AECs autophagy. Human AECs were treated with or without TNF‐α. Our data showed that the combination of GLCCI1 and WDR45B was partly inhibited in TNF‐α‐stimulated human AECs (Figure 3A). In protein synthesis inhibitor CHX‐treated human AECs infected with LV‐NC, the expression of WDR45B was obviously inhibited at 4 hours after CHX stimulation. However, in GLCCI1‐overexpressed human AECs, the expression of WDR45B was significantly inhibited at 1 hour after CHX stimulation (Figure 3B). Overexpression of GLCCI1 could significantly enhance the inhibitory effect of CHX on WDR45B synthesis. Besides, we further revealed that increasing of GLCCI1 suppressed WDR45B expression and decreasing of GLCCI1 promoted WDR45B expression (Figure 3C). Importantly, we indicated that increasing of GLCCI1 reduced Beclin‐1 and LC3‐II/LC3‐I expression and raised p62 expression in TNF‐α‐treated human AECs, while WDR45B overexpression could significantly reverse the influence of GLCCI1 (Figure 3D). Our results showed that the expression of WDR45B can be significantly increased by LV‐WDR45B infection (Figure S1). In addition, the inhibition of GLCCI1 to TNF‐α‐induced autophagosome also was proved to be partly reversed by increasing of WDR45B (Figure 3E). Our data revealed that GLCCI1 inhibited autophagy in human AECs through inhibition of WDR45B.

### 3.4 GLCCI1 inhibited mouse AECs autophagy through binding to WDR45B

**FIGURE 4:**
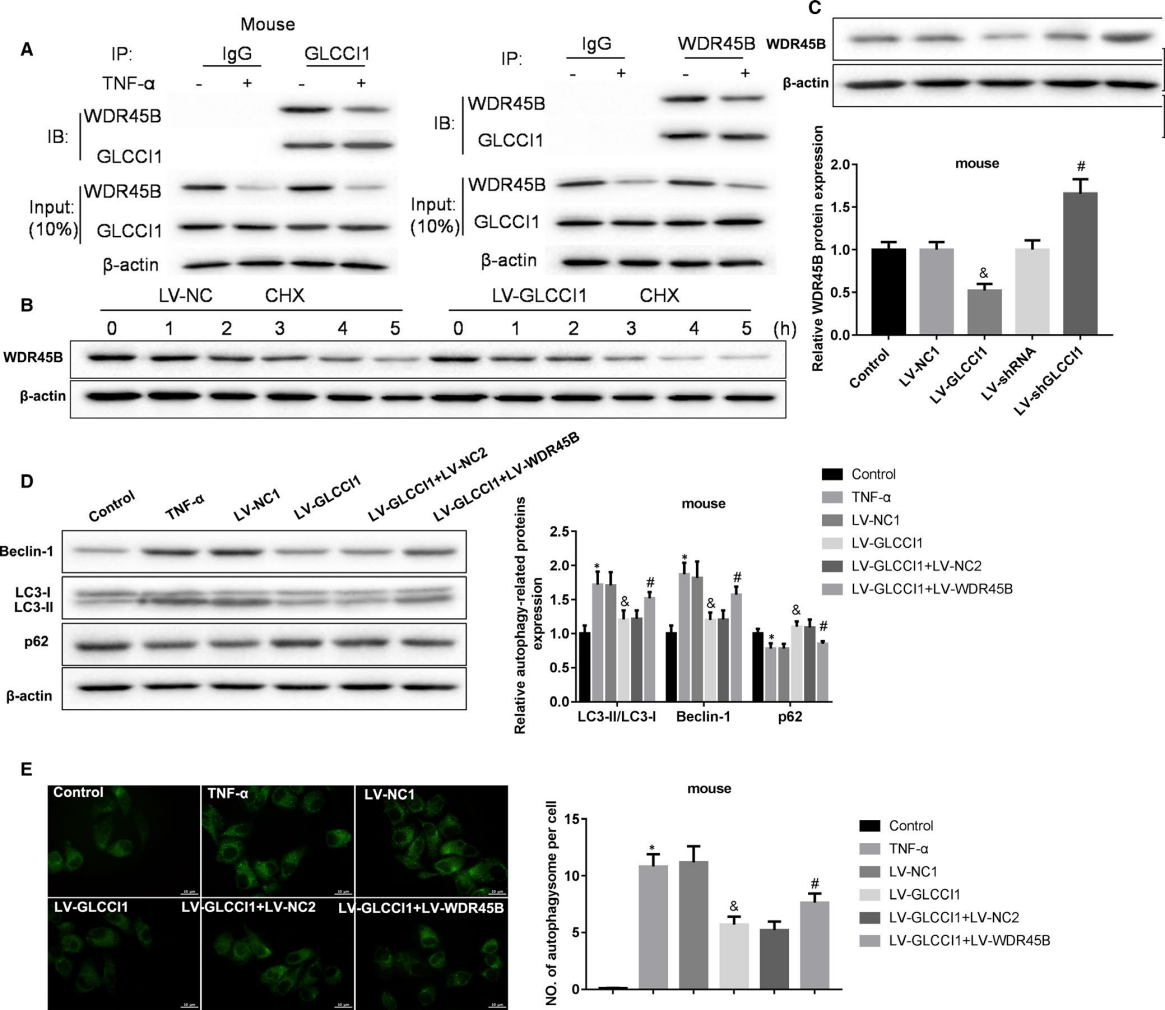
GLCCI1 inhibited TNF‐α‐induced mouse AECs autophagy by binding with WDR45B and suppression its expression. A, Co‐IP experiments were carried out to examine the combination between GLCCI1 and WDR45B. B, 50 mg/mL of CHX was used to stimulate human AECs infected with LV‐NC or LV‐GLCCI1 for 0, 1, 2, 3, 4 and 5 h. Then, the expression of WDR45B in cells was measured by Western blotting assay. C, Western blotting assay was performed to examine the expression of WDR45B in mouse AECs infected with LV‐GLCCI1 or LV‐shGLCCI1. N = 3. ^&^ *P* < .05 contrasted with LV‐NC1 group, and ^#^ *P* < .05 compared with LV‐shRNA group. D, Western blotting assay was implemented to detect the expression of Beclin‐1, LC3‐II, LC3‐I and p62 in mouse AECs co‐infected LV‐GLCCI1 with or without LV‐WDR45B. E, Formation of autophagosome was measured using immunofluorescence assay. N = 3. *P < .05 compared with control group, ^&^ *P* < .05 contrasted with LV‐NC1 group, and ^#^ *P* < .05 compared with LV‐GLCCI1 group

In order to confirm the above results are also applicable to animal experiments, we tested the relationship between GLCCI1 and WDR45B in mouse AECs. The results also showed that TNF‐α weakened the combination of GLCCI1 and WDR45B in mouse AECs (Figure 4A). Overexpression of GLCCI1 enhanced the inhibitory effect of CHX on WDR45B production (Figure 4B). Besides, overexpression of GLCCI1 impeded WDR45B expression, and knockdown of GLCCI1 promoted WDR45B expression (Figure 4C). Furthermore, in TNF‐α‐stimulated mouse AECs, we also demonstrated that increasing of WDR45B could significantly restrain the inhibitory effect of GLCCI1 on Beclin‐1 and LC3‐II/LC3‐I expression and autophagosome formation, and the promotory effect of GLCCI1 on p62 expression (Figure 4D,E). All in all, we proved that GLCCI1 inhibited TNF‐α‐induced autophagy in mouse AECs via binding to WDR45B and inhibiting WDR45B expression.

### 3.5 GLCCI1 improved airway remodelling in ovalbumin‐sensitized mice via inhibition of WDR45B

**FIGURE 5:**
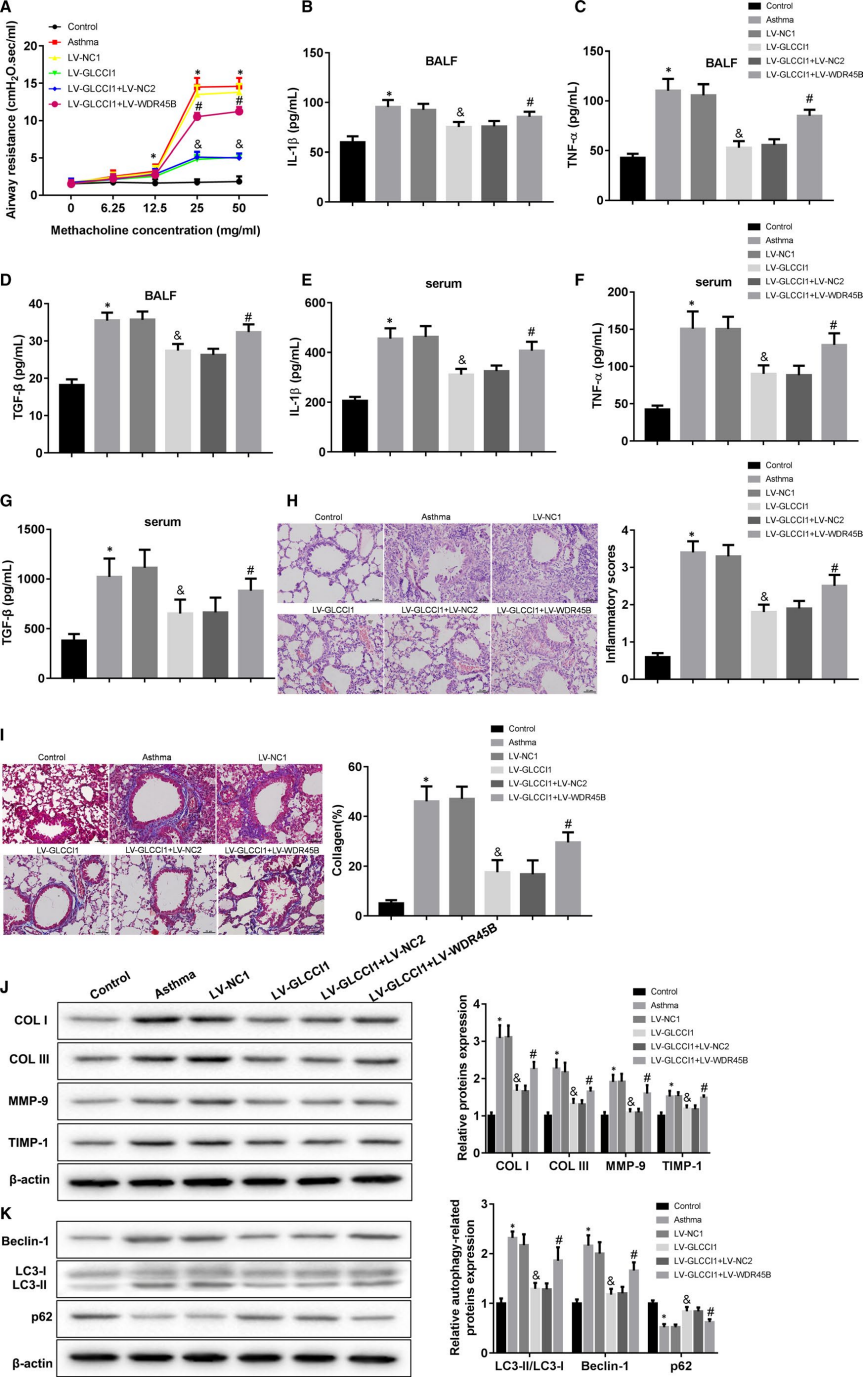
Effect of GLCCI1 on airway remodelling in asthma. A, All mice were stimulated with methacholine at increasing of doses, and then, the airway resistance of mice was examined. B‐D, The concentration of IL‐1β, TNF‐α and TGF‐β in BALF of mice was measured by ELISA. E‐G, The concentration of IL‐1β, TNF‐α and TGF‐β in serum of ovalbumin‐sensitized mice was also detected using ELISA. H, H&E staining was performed to examine the pathological changes in lung tissues of mice, and the inflammatory score of lung tissues was analysed. I, Masson staining was carried out to measure the deposition of collagen in lung tissues of mice. J and K, Western blotting assay was implemented to examine the expression of COL I, COL III, MMP‐9, TIMP‐1, Beclin‐1, LC3‐II, LC3‐I and p62 in lung tissues of mice. N = 6 mice per each group. *P < .05 compared with control group, ^&^ *P* < .05 contrasted with LV‐NC1 group, and ^#^ *P* < .05 compared with LV‐GLCCI1 + LV‐NC2 group

Then, we explore the effect of GLCCI1 on airway remodelling in ovalbumin‐sensitized mice. Our results demonstrated overexpression of GLCCI1 could effectively down‐regulate the airway resistance in ovalbumin‐sensitized mice, while increasing of WDR45B partly reversed the down‐regulated airway resistance (Figure 5A). The inhibitory effect of GLCCI1 on IL‐1β (Figure 5B), TNF‐α (Figure 5C) and TGF‐β (Figure 5D) production in the BALF of ovalbumin‐sensitised mice also was partly reversed by overexpression of WDR45B. Consistently, the inhibition of GLCCI1 to IL‐1β (Figure 5E), TNF‐α (Figure 5F) and TGF‐β (Figure 5G) production in the serum of ovalbumin‐sensitized mice as well as partly rescued by increasing of WDR45B. Moreover, H&E staining showed that a large number of immune cells infiltrated in the lung tissues of ovalbumin‐sensitised mice. Overexpression of GLCCI1 notably reduced the inflammatory response in ovalbumin‐sensitized mice, while it was reversed by increasing of WDR45B (Figure 5H). The deposition of collagen fibre in lung tissues also was reduced by overexpression of GLCCI1, but increasing of WDR45B obviously inhibited the effect of GLCCI1 (Figure 5I). Furthermore, COL I, COL III, MMP‐9 and TIMP‐1 expression was promoted in the lung tissues of ovalbumin‐sensitized mice, and overexpression of GLCCI1 could significantly suppress the expression of these factors. However, the inhibitory effects of GLCCI1 on these factors were rescued by increasing of WDR45B (Figure 5J). The inhibition of GLCCI1 to LC3‐II/LC3‐I and Beclin‐1 expression in the lung tissues of ovalbumin‐sensitized mice, and promotion to p62 expression were partly reversed by increasing of WDR45B (Figure 5K). Overall, our data demonstrated that GLCCI1 impeded the airway resistance in ovalbumin‐sensitized mice via inhibition of WDR45B.

## 4 DISCUSSION

Asthma is a serious public health problem in the world and reduces the quality of life of patients. Now, more and more researchers focus their study on exploring the pathogenesis of asthma. Hence, a mature animal asthma model is very important. Ovalbumin is a classical drug that was used to establish mouse asthma model in many studies.^19^ Here, our data showed that the airway resistance in ovalbumin‐stimulated mice was significantly higher than normal mice. Besides, highly expressed pro‐inflammatory cytokines, damaged lung tissues and excessively deposited collagen were found in ovalbumin‐induced mice, indicating that we established mouse asthma model using ovalbumin successfully. Importantly, we demonstrated that the expression of GLCCI1 was suppressed, autophagy was activated in lung tissues of ovalbumin‐sensitized mice. In this present study, we explored the mechanism of GLCCI1 in regulating asthma development through activation autophagy via binding with WDR45B.

As an important regulator in cell homeostasis, autophagy was proved to be related with almost all disorders, such as cancer, neurodegenerative disorders, amyotrophic lateral sclerosis, atherosclerosis and asthma.^20^, ^21^, ^22^, ^23^ Although autophagy routinely plays a protective role, the self‐cannibalistic, paradoxically, even the prosurvival functions of autophagy may be deleterious in certain experimental disease setting.^24^ For instance, Xia et al^25^ reported that interleukin 4 could aggravate asthma through activation the autophagy in pulmonary B cells via regulating mTOR signalling and Ptdlns3K signalling. Gu et al^26^ demonstrated that simvastatin effectively improves the airway remodelling and inflammation in mouse asthma model through activation of the autophagy of bronchial smooth muscle cells. These data revealed that autophagy is an enhancer of asthma and may also is a suppressor in asthma development. Autophagy was performed by a series of proteins. During this process, the soluble form LC3‐I changes into lipidated form LC3‐II under the regulation of ATG4. The conversion of LC3‐I to LC3‐II was considered as a major feature of the formation of autophagosome.^27^, ^28^ Moreover, p62 is an ubiquitin‐binding protein that targets and binds to other proteins for selective autophagy, and these proteins are ultimately degraded in the lysosome.^29^ It was demonstrated that p62 accumulates when autophagy is inhibited, and inversely, concentrations of p62 decrease when autophagy is induced.^13^ Our data indicated that overexpression of GLCCI1 suppressed, whereas down‐regulation of GLCCI1 promoted autophagy‐related proteins expression and formation of autophagosome in human and mouse AECs. In vivo, the data showed that overexpression of GLCCI1 notably declined the airway resistance, reduced the production of pro‐inflammatory cytokines and improved the damage of lung tissues in ovalbumin‐sensitised mice. Importantly, overexpression of GLCCI1 inhibited the activation of autophagy in ovalbumin‐sensitized mice.

WDR45B is a member of WIPI protein family universally expressed in human tissues. WDR45B is a marker of autophagy. It was indicated that WDR45B and WIPI4 fold into 7‐bladed β‐propeller protein, and then, this protein bind with PtdIns3P followed by co‐localization at autophagosome.^30^ Furthermore, Bakula et al also recognized that the 7‐bladed β‐propeller protein formed by WDR45B together with WIPI4 is a scaffolds for liver kinase B1‐AMP‐activated protein kinase‐tuberous sclerosis complex during autophagy.^16^ In our present study, we found the combination between WDR45B and GLCCI1. Both in human and mouse AECs, increasing of GLCCI1 inhibited WDR45B expression, while decreasing of GLCCI1 facilitated WDR45B expression. Furthermore, our results indicated that increasing of WDR45B reversed the inhibition of GLCCI1 increasing to autophagy in human and mouse AECs in vivo, and the improvement of GLCCI1 increasing on lung damage in vitro. Importantly, the inhibition of GLCCI1 to autophagy in the lung tissues of ovalbumin‐sensitized mice was reversed by WDR45B increasing.

Overall, our data indicated that GLCCI1 could effectively improve the airway remodelling in ovalbumin‐sensitized mice through inhibiting asthma‐induced autophagy via combination with WDR45B and suppressing its expression. Our data proved a novel pathogenesis of asthma and provided a potential target for asthma therapy.

## CONFLICTS OF INTEREST

The authors confirm that there are no conflicts of interest.

## AUTHOR CONTRIBUTION

**Qiufen Xun:** Conceptualization (equal); Funding acquisition (equal); Investigation (equal); Project administration (equal); Writing‐original draft (equal); Writing‐review & editing (equal). **Jiulong Kuang:** Data curation (equal); Investigation (equal); Methodology (equal). **Qing Yang:** Investigation (equal); Resources (equal); Software (equal). **Wei Wang:** Formal analysis (equal); Investigation (equal). **Guofeng Zhu:** Formal analysis (equal); Validation (equal); Visualization (equal).

## Supporting information

Fig S1

## Data Availability

The data that support the findings of this study are available from the corresponding author upon reasonable request.
