# Supplementary figures and images for: GLCCI1 reduces collagen deposition and airway hyper‐responsiveness in a mouse asthma model through binding with WD repeat domain 45B
Source: J Cell Mol Med. 2021 May 28;25(14):6573–83. doi: 10.1111/jcmm.16658 (PMC8278071; doi:10.1111/jcmm.16658)

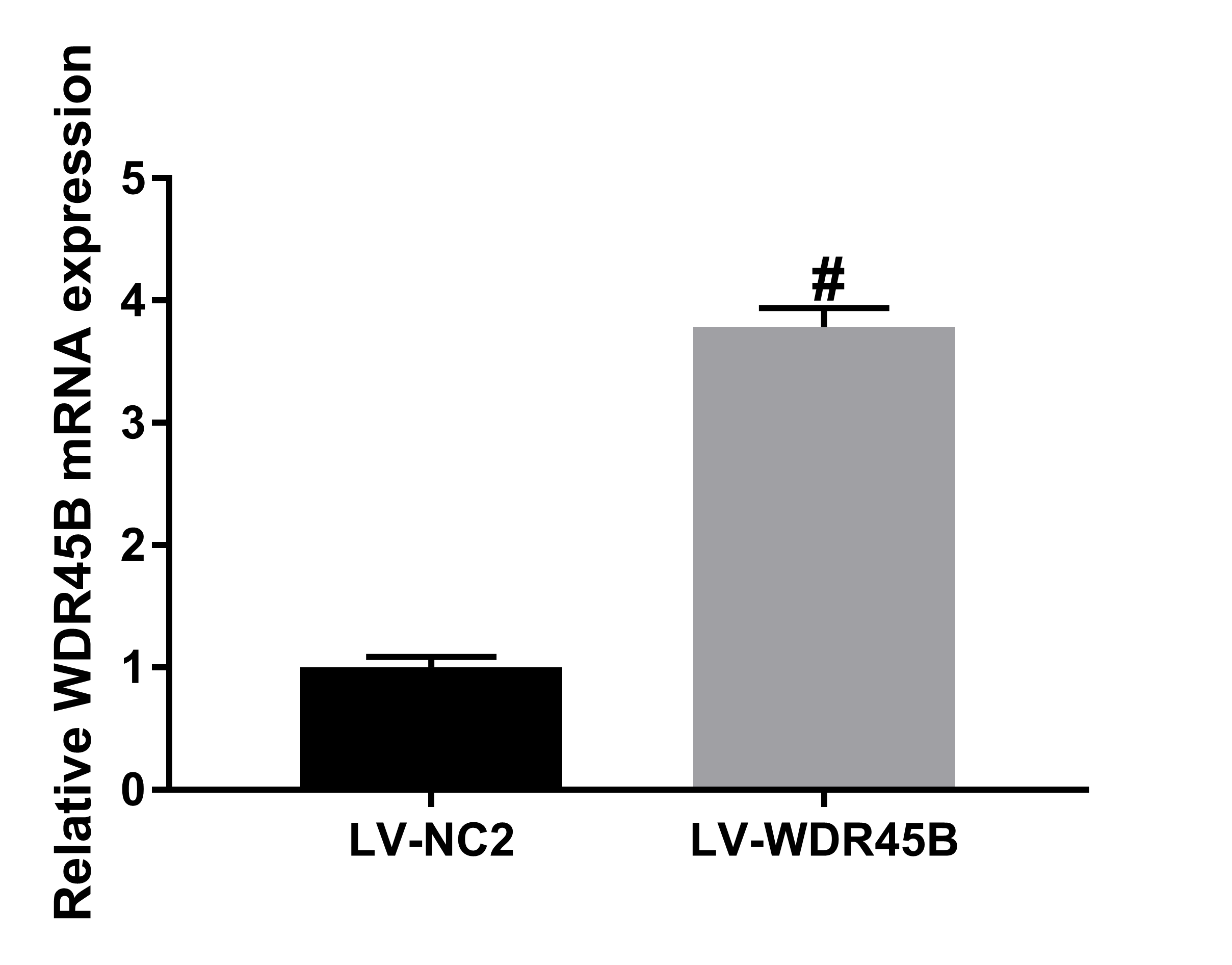

Supplement: Supplementary file 1 — Fig S1 [file JCMM-25-6573-s001.tif]
